# Risk factors of oesophageal cancer at health facilities in Addis Ababa, Ethiopia: Unmatched case control study

**DOI:** 10.3389/fonc.2022.997158

**Published:** 2022-09-20

**Authors:** Berhe Dessalegn, Fikre Enqueselassie, Mirgissa Kaba, Mathewos Assefa, Adamu Addissie

**Affiliations:** ^1^ Department of Public Health, College of Medicine and Health Sciences, Adigrat University, Adigrat, Ethiopia; ^2^ Department of Preventive Medicine, School of Public Health, College of Health Sciences, Addis Ababa University, Addis Ababa, Ethiopia; ^3^ Department of Radiotherapy Center, School of Medicine, Addis Ababa University, Addis Ababa, Ethiopia

**Keywords:** oesophageal cancer, risk factors, case control, positive predictors, ethiopia

## Abstract

**Background:**

Oesophageal carcinoma is one of the most common cancers in Ethiopia. Its occurrences vary among regional states of the country. The identification of local risk factors of oesophageal cancer will make it simple to design a focused intervention. On local risk factors, there is, however, a shortage of empirical evidence. Therefore, the aim of study was to identify local risk factors.

**Methods:**

An unmatched case control study design was employed. From February 2019 to August 2020, 338 histologically confirmed cases and 338 controls were recruited consecutively from six health facilities in Addis Ababa, Ethiopia’s capital city. To collect data from the cases and the controls, face to face interviews were conducted. Epi-info version 7 was used to enter and cleaned data, and SPSS version 23 was used to analyze it. The odds ratio was calculated based on hierarchal model multivariable logistic regression, and statistically significance was declared at p-value of <0.05.

**Results:**

The mean (SD) age of the cases and the controls was 54.3 ± 12.5 years old and 40.2 ± 13.7 years old, respectively. The odds of oesophageal cancer was significantly higher among older ages (OR =11.0, 95% CI [6.60, 20.91]), rural residents (OR = 4.2, 95% CI [1.04, 16.80]), and those who had history of smoking (OR =1.3, 95% CI [1.12, 1.60]), khat chewing (OR = 4.0, 95% CI [2.50, 6.60]), raw meat consumers (OR = 2.6, 95% CI [1.75, 3.90]). Increasing monthly income (OR = 0.2, CI 95% [0.09, 0.49]) and a habit of eating fruits or vegetables (OR = 0.49, 95% CI [0.32, 0.76]) were associated with lower risks.

**Conclusions:**

Tobacco smoking, khat chewing, age, residency, and red raw meat consumption were discovered to be positive predictors of oesophageal cancer, whereas fruit or vegetable consumption and higher monthly income were discovered to be inversely associated. It is advised to avoid the use of khat and tobacco, as well as to avail fruits and vegetables in dish.

## Introduction

Cancer is now the leading cause of morbidity and mortality among non-communicable diseases worldwide. In 2020, there were 19.3 million new cancer cases and 10 million cancer deaths worldwide. It is currently putting enormous strain on the health-care system and the global economy (1).

The Esophagus is a 25.4-centimeter-long organ in the digestive system that transports food and drink from the mouth to the stomach. Oesophageal cancer begins in the esophagus cells. The two most common histological subtypes are oesophageal squamous carcinoma and oesophageal adenocarcinoma (2).

Oesophageal squamous carcinoma is still the most common subtype worldwide. In 2018, an estimated 572,000 new cases of oesophageal cancer were diagnosed worldwide, with oesophageal squamous carcinoma accounting 85% whereas oesophageal adenocarcinoma and other subtypes accounted 15% (3).

In 2020, oesophageal cancer was the eighth most common cancer and the sixth leading cause of cancer deaths globally (4). Oesophageal cancer is more common in poor countries, and it is the third most common gastrointestinal cancer and the fourth most common cancer overall (5). In addition, in Sub-Saharan Africa, oesophageal cancer is characterized by poor patients prognosis and survival rate (6).

In developed countries, the adenocarcinoma subtype has surpassed squamous carcinoma (3, 4). Meanwhile, in Sub-Saharan Africa, the prevalence of oesophageal cancer varies by country. The disparity could be explained by underlying oesophageal cancer risk factors. In developing countries, oesophageal squamous carcinoma is common, whereas oesophageal adenocarcinoma is common in Western countries (7).

Some risk factors for the development of oesophageal cancer have been identified in the literature, such as age, tobacco smoking, and a lack of certain micronutrients for squamous carcinoma, and Barrett’s oesophagus and obesity for adenocarcinoma (4, 8). Barrett’s esophagus was linked with an increased risk of developing adenocarcinoma (9). Other cancers, such as lung, head, neck and colorectal cancers also increased the risk of oesophageal cancer (10).

According to some hospital reports, the incidence of oesophageal cancer has been increasing in Ethiopia over the last few decades. As a result, evidence on local risk factors is critical for reducing and/or mitigating oesophageal cancer-related morbidity and mortality in the country. Nonetheless, there is insufficient evidence on local risk factors for oesophageal cancer in Ethiopia. A small number of studies were conducted, but they were either limited to a specific area (11) or focused on single risk factor of oesophageal cancer (12–14). Thus, herein, taking into account the gaps from the previous studies, we were motivated to carry out this important study in selected health facilities of Addis Ababa to identify the local risk factors of oesophageal cancer in Ethiopia.

## Materials and methods

### Study design

In Addis Ababa, Ethiopia’s capital city, an unmatched case-control study design was used. For our research, we selected six health care facilities. The study facilities were selected based on case load, infrastructure availability, diversity of health professionals, and previous track records on cancer diagnosis and treatment services. In Addis Ababa, there were 11 public and 33 private hospitals. In addition, 88 public and six non-governmental health centers, as well as 777 private clinics, served residents of the capital as well as patients from all over Ethiopia (15). The cases were all adult patients who were histologically and clinically confirmed to have oesophageal cancer, while the controls were all adults who were endoscopically ruled out of having oesophageal cancer and resulted negative for oesophageal cancer. The study included 338 people aged 18 and up who were diagnosed with oesophageal cancer in Addis Ababa’s selected health facilities between February 2019 and August 2020. In addition, 338 healthy controls were included in the study, with endoscopy confirming that they did not have oesophageal cancer. The controls were recruited from the same health facilities and over the same time period as the patients who were referred to the gastroenterology department for an endoscopy and were found to be cancer-free.

The sample size was calculated using the double population proportion formula. As a result, the proportion of cases (P1) and controls (P2) who smoked cigarettes was calculated as p1 = 46% and p2 = 32%, respectively (16). The researchers chose the cases and the controls ratio of r = 1:1, Z/2 = level of confidence = 1.96, Z = power of the study = 0.8, and a non-response rate of 5%. As a result, we had ended up with 296 participants, but to increase the precision of the estimates, we included all available cases and controls aged 18 years and older (n=676).

The questionnaire was developed and contextualized to the local conditions after reviewing various literatures. Variables included in the questionnaire were socio-demographic, socio-economic, behavioral, dietary, and lifestyle information. The questionnaire was first prepared in English language and then translated it in to Amharic (the national working language) by language experts and back to English to ensure consistency and understandability of the tool for both data collectors and study participants. We recruited eleven data collectors with a BSc in nursing and experienced in cancer-related research activities, as well as one oncology resident to serve as supervisor. Both the supervisor and the data collectors were trained for two days on the purpose of the study and the importance of quality data. Data were collected through face-to-face interviews with study participants.

Epi-Info software version 7.0 was used to code, enter, and clean data before exporting it to SPSS software version 23 for analysis. Initially, descriptive statistics on socio-demographic, socio-economic, behavioural, dietary and other variables were conducted. Numerical data were summarized using mean and standard deviation (SD) for symmetrical data or median and inter quartile range (IQR) for skewed data, whereas categorical data were summarized using frequency and presented in texts and tables.

Cross tabulation was used to examine the crude association of each predictor variable with the outcome variable. The predictor variables were then identified using multiple variable hierarchical logistic regression analysis models. Model I included socio-demographic and socio-economic variables, Model II included socio-demographic and behavioral variables, Model III included socio-demographic, behavioral, and hot drinks variables, and Model IV included socio-demographic, behavioral, hot drink, and diet related variables. A p-value of 0.1 was used as a cut point for variables to be included in subsequent models and then to the final multivariable logistic regression based on experts’ opinion. Odds ratios with 95% confidence intervals were calculated for each predictor variable versus the outcome variable or oesophageal cancer status. A statistically significance was declared at p-value of< 0.05.

## Results

### Socio-demographic and socio-economic characteristics of cases and controls

Of the 702 cases and controls approached for participation, 676 (96.3%) of them provided their responses. The cases and controls had mean (standard deviation) ages of 54.32 ( ± 12.5) and 40.87 ( ± 13.7), respectively. More than half (52%) of the cases were over the age of 55, but this was only true for less than a quarter (20.4%) of the controls. Males constituted more than half of the cases (52.4%) and 55.6% of the controls. Approximately 63% of the cases and 68% of the controls came from Ethiopia’s rural and urban areas, respectively. More than six out of ten (61.8%) of the cases were unable to read and write, whereas only about three in ten (30.0%) holds true for the controls group.

The cases and the controls had mean (standard deviation) monthly income of 64 ( ± 59) and 91.79 ( ± 67) USD, respectively. About 89% of the cases and 83% of the controls were recruited from Tikur Anbesa Specialized Hospital, respectively. For the cases and the controls, the median (Interquartile range) distance from home to the diagnostic/treatment center was 350 (200 to 560) and 280 (30 to 500) kilometers, respectively. Approximately 72% of the cases and 63% of the controls paid for their medical expenses out of their pocket Most of the cases (51.8%) belonged to the Muslim faith, and 37.6% of them were farmers, which made up the majority of the cases’ occupations (**Table 1**).

**Table 1:** Socio-demographic characteristics of cases and controls Addis Ababa, Ethiopia, from February 2019 to August 2020.

Variables	Cases frequency (%)	Controls frequency (%)	Total
**Age categories**			
<35	24 (7)	147 (43.4)	171
35-44	46 (14)	62 (18.4)	108
45-54	91 (27)	60 (17.8)	151
≤55^+^	177 (52)	69 (20.4)	246
**Gender**			
Male	177 (52.4)	188 (55.6)	365
Female	161 (47.6)	150 (44.4)	311
**Religion**			
Christianity	159 (47)	240 (71)	399
Islam	175 (51.8)	97 (28.7)	272
Wakefata	4 (1.2)	1 (0.3)	5
**Residency**			
Urban	126 (37.3)	231 (68.3)	357
Rural	212 (62.7)	107 (31.7)	319
**Educational status**			
Unable to read and write	209 (61.8)	96 (28.4)	305
Grade 1-8	72 (21.3)	99 (29.3)	171
Grade 9-12	37 (10.9)	83 (24.6)	120
Diploma and above	20 (5.9)	60 (17.8)	80
**Occupation**			
Government workers	38 (11.2)	57 (16.9)	95
House wife (Homemaker)	118 (34.9)	78 (23.1)	196
Merchant	20 (5.9)	66 (19.5)	86
Private worker	35 (10.4)	77 (22.8)	112
Farmer	127 (37.6)	60 (17.8)	187
**Current marital status**			
Married	246 (72.8)	170 (50.3)	416
Single	92 (27.2)	168 (49.7)	260
**Family size**			
<=2	16 (4.7)	62 (18.3)	78
3-5	152 (45.0)	196 (58.0)	348
>=6	170 (50.3)	80 (23.7)	250
**Monthly income (USD)**			
<35	171 (50.6)	89 (26.3)	260
35-106	130 (38.5)	149 (44.1)	279
106.6-177	21 (6.2)	71 (21)	92
>177	16 (4.7)	29 (8.6)	45
**Median distance to Addis Ababa in kilometers (IQR)**	350 (200,560)	180 (15, 382)	280 (30,500)

### Behavioral characteristics of cases and controls

Tobacco use was prevalent in 22.2% of the cases and 5% of the controls, respectively. Meanwhile, 15% of the cases and 1.2 percent of the controls had smoked for 15 years or more. The prevalence of alcoholic consumption was 32% in the cases and 24.0% in the controls, respectively. Approximately 12% of ever drinker cases testified that they consumed alcohol on a daily basis, but this was only reported on 1.5% of ever drinker controls. Only 1.8% of the ever drinker controls had experienced memory loss as a result of alcohol consumption, whereas 11% of the ever drinker cases had such experiences.

The mean (standard deviation) of alcohol consumption per day among the cases and the controls was 4.3 ( ± 2.4) and 2.4 ( ± 1.2), respectively. Khat chewing was reported in 36.7% of the cases and 12.1% of the controls. Meanwhile, 32.5% of the cases and 1.8% of the controls had chewed khat for 10 years or more, respectively. The mean (standard deviation) of chewing khat bundles per week in the cases and the controls were 3.1( ± 1.5) and 1.7 ( ± 1.1), respectively. Approximately 14.5% of the cases and 3.0% of the controls smoked tobacco while chewing khat, respectively. Furthermore, 7.4% of the cases and 1.8% of the controls consumed alcohol, tobacco, and khat at the same time (**Table 2**).

**Table 2:** Behavioral characteristics of cases and controls, Addis Ababa, Ethiopia, February 2019-August 2020.

Variables	Cases frequency (%)	Controls frequency (%)	Total
**Tobacco smoking status**			
Never smokers	263 (77.8)	321 (95)	584
Ever smokers	75 (22.2)	17 (5)	92
**Years of tobacco smoking**			
<15 years	23 (6.8)	12 (3.8)	35
15^+^ years	51 (15.1)	5 (1.2)	56
**Pieces of tobacco used per day**			
<15 pieces of cigarettes	56 (16.6)	16 (4.7)	72
15^+^ pieces of cigarettes	17 (5.0)	1 (0.3)	18
**Ever consumed any alcoholic beverages**			
Yes	105 (31.1)	80 (23.7)	185
No	233 (68.9)	258 (76.3)	491
**Number of alcohol drinks per day(unit)**			
1-2	43 (12.7)	34 (10.1)	77
3-4	35 (10.4)	30 (8.8)	65
5+	27 (8.0)	16 (4.9)	43
**Experiences of memory loss due to alcohol**			
Yes	35 (10.4)	6 (1.8)	41
No	70 (20.7)	74 (21.9)	144
**Ever chewed khat**			
Yes	124 (36.7)	41 (12.1)	165
No	214 (63.3)	297 (87.9)	511
**Years of chewing khat**			
<10 years	14 (4.1)	35 (10.4)	49
10^+^ years	110 (32.5)	6 (1.8)	116
**Mean bundle of khat consumed per week (SD)**	3.1 (1.5)	1.7 (1.1)	2.7 (1.5)
Tobacco and alcohol consumption	40 (11.8)	10 (3)	50
Tobacco and khat consumption	49 (14.5)	10 (3.0)	59
Alcohol and khat consumption	37 (10.9)	17 (5)	54
Tobacco, alcohol and khat consumption	25 (7.4)	6 (1.8)	31

### Hot beverage and dietary intake characteristics of the cases and the controls

About ten percent of the cases and six percent of the controls reported drinking two or more cups of hot black tea per day. Two cups of tea with milk or more per day were reported by 6.5 percent of the cases and 1.2 percent of the controls. Furthermore, two cups or more of coffee per day were common among 50 percent of the cases and 41.7 percent of the controls. Teff was the most popular staple diet among both the cases and the controls, but sorghum was the second most popular staple diet among 14% of the cases. The proportion of the cases who consumed hot porridge (25 percent) was higher than the proportion of the controls (2.4 percent). Despite the fact that nearly 14 percent of the cases reported eating food at very high temperatures, only 9 percent of the controls reported similar experiences. Consumption of red raw meat was reported by 55 percent of the cases, but only 28 percent of the controls reported doing so. Consumption of fresh fruits and vegetables per day was common in 28.4 percent of the controls but less common or in 21.6 percent of the cases (**Table 3**).

**Table 3:** Hot beverage and dietary characteristics of cases and controls February 2019-August 2020, Addis Ababa, Ethiopia.

Variables	Cases frequency (%)	Controls frequency (%)	Total
**Hot black tea consumption per day**			
≤2 cups	306 (90.5)	318 (94.1)	624
2+ cups	32 (9.5)	20 (5.9)	52
**Hot tea with milk consumption per day**			
≤2 cups	316 (93.5)	334 (98.8)	650
2+ cups	22 (6.5)	4 (1.2)	26
**Hot coffee consumption per day**			
≤2 cups	169 (50)	197 (58.3)	366
2+ cups	169 (50)	141 (41.7)	310
**Hot spice tea consumption per day**			
≤ 2 cups	27 (8)	9 (2.7)	640
2+ cups	311 (92)	329 (97.3)	36
**Temperature of hot drink**			
Normal	150 (44.4)	178 (52.7)	328
Hot	147 (43.4)	130 (38.5)	277
Very hot	41 (12)	30 (8.9)	71
**Most common diet (Staple diet)**			
Teff	201 (60)	230 (68)	431
Sorghum	46 (14)	33 (9.8)	79
Maize	29 (8.6)	28 (8.3)	57
Wheat	35 (10.3)	34 (10)	69
Barley	13 (3.8)	8 (6.8)	21
Others*	14 (4.1)	5 (1.5)	19
**Category of food preparation**			
Injera	215 (64)	300 (88.8)	515
Bread	12 (3.6)	18 (5.3)	30
Porridge	86 (25.0)	8 (2.4)	94
Broth	12 (3.6)	5 (1.5)	17
Others**	13 (3.8)	7 (2.0)	20
**Food temperature**			
Normal	203 (60.1)	218 (64.5)	421
Hot	88 (26)	92 (27.2)	180
Very hot	47 (13.9)	28 (8.3)	75
**Ever consumed raw meat**			
Yes	186 (55)	94 (27.8)	280
No	152 (45)	244 (72.2)	396
**Fruit or vegetable consumption**			
Yes	73 (21.6)	96 (28.4)	169
No	265 (78.4)	242 (71.6)	507

Others* = False banana, small millet.

Others** = “Kita”.

### Comorbidity profile of the cases and the controls

The clinical data of 11.2 percent of the cases and 6.2 percent of the controls revealed a history of diabetes mellitus. Furthermore, 11.8 percent of the cases and 6.2 percent of the controls had a first-degree family history of any type of cancer. Oesophageal cancer was ranked the second among the cancers reported by both cases and controls. Furthermore, 2.4 percent of the cases and 1.8 percent of the controls had the first-degree family history of oesophageal cancer. About 60.4 percent of the controls and 50 percent of the cases had a history of persistent helicobacter pylori infection (**Table 4**).

**Table 4:** Comorbidity profile of cases and controls, February 2019-August 2020, Addis Ababa, Ethiopia.

Variables	Cases frequency (%)	Controls frequency (%)	Total
**Previous diagnosis of DM**			
Yes	38 (11.2)	21 (6.2)	59
No	300 (88.8)	317 (93.8)	617
**Types of DM**			
DM type 1	9 (2.7)	6 (1.8)	15
DM type 2	29 (8.6)	14 (4.1)	43
Gestational DM	0 (0)	1 (0.3)	1
**First degree family history of any cancer**			
Yes	40 (11.8)	21 (6.2)	61
No	298 (88.2)	317 (93.8)	615
**Types of cancer**			
Breast cancer	20 (6.0)	3 (0.9)	23
Esophageal cancer	8 (2.4)	6 (1.8)	14
Liver cancer	7 (2.1)	3 (0.9)	10
Gastric cancer	5 (1.5)	5 (1.5)	10
Others	2 (0.6)	2 (0.6)	4
**Relationship**			
Mother	12 (3.6)	5 (1.5)	17
Father	7 (2.1)	9 (2.7)	16
Sister	9 (2.7)	4 (1.2)	13
Brother	12 (3.6)	3 (0.9)	15
**Previous helicobacter pylori infection**			
Yes	169 (50)	204 (60.4)	374
No	169 (50)	134 (39.6)	303

Participants who had the habit of both tobacco smoking and alcohol drinking had 7.1 (AOR = 7.1, 95% CI [2.94, 16.94]) times higher risk/odds to develop oesophageal cancer compared to those who had no habit of smoking and alcohol drinking. Likewise, the odds/risks of oesophageal cancer development increased by 10.1 times among both tobacco smokers and khat chewers (AOR = 10.1, 95% CI [4.3, 23.4]) compared to those who used neither tobacco nor khat.

The study also revealed that consumptions of all tobacco, khat and alcohol had been alarmingly increasing the odds/risks of oesophageal cancer development by 11.6 times (AOR = 11.6, 95% CI [3.93, 34, 53]) compared to those who used neither of them. Furthermore, as one year increased in khat chewing, the likelihood of oesophageal cancer development had increased by 1.4 times (OR = 1.4, 95% CI [1.21, 1.57]).

Age is an important factor for oesophageal cancer development, thus, according to our study, individuals at the age of 55 years or more were 11.0 times higher to develop esophageal cancer compared to individuals below the age of 35 years (AOR = 11.0,95% CI [6.60,20.91]). Moreover, the odds of oesophageal cancer development increased by 4.2 fold among rural residents compared to the urban ones (AOR = 4.2, 95% CI [1.04, 16.8]).

The odds/risks of oesophageal cancer was 1.2 times higher among individual who used to consume hot coffee greater than 2 times per day compared to those consumed hot coffee per day less than 2 times (AOR = 1.2,95% CI [1.12,4.64]). Ever consumption of red raw meat increased the odds/risks of oesophageal cancer by 2.6 times (AOR = 2.6, 95% CI [1.75, 3.90]) compared to their counter parts. However, fruit and vegetable consumptions on daily bases decreased the odds/risk of oesophageal cancer by at most 68% or at least 24% (AOR = 0.49, 95% CI [0.32, 0.76]) in other words, fruit and vegetable consumptions on daily bases was inversely associated with esophageal cancer development (**Table 5**).

**Table 5:** Statistically significant variables associated with oesophageal cancer based on hierarchical modeling from February 2019 to August 2020, Addis Ababa, Ethiopia.

Variable	Cases (n %)	Controls (n %)	COR (95%CI)	P-value	AOR (95% CI)	P-value
**Tobacco and alcohol**						
None of them	198 (56.6)	251 (74.3)	Ref			
One of them	100 (29.6)	77 (22.8)	1.6 (1.16, 2.34)	0.005	1.7 (1.11, 2.68)*	0.016
Both	40 (11.8)	10 (3)	5.1 (2.47, 10.39)	0.0001	7.1 (2.94, 16.94)*	0.0001
**Tobacco and khat**						
None of them	188 (55)	290 (85.8)	Ref.			
One of them	101 (29.9)	38 (11.2)	4.1 (2.71, 6.21)	0.0001	5.6 (3.23,9.69)*	0.001
Both of them	49 (14.5)	10 (3.0)	7.56 (3.74,15.29)	0.0001	10.1 (4.3,23.4)*	0.0001
**Alcohol and khat**						
None of them	146 (43.2)	234 (69.2)	Ref.			
One of them	155 (45.9)	87 (25.7)	2.8 (2.04, 3.9)	0.0001	2.8 (1.85,4.34)*	0.0001
Both of them	37 (10.9)	17 (5)	3.5 (1.89, 6.42)	0.0001	5.3 (2.45,11.39)*	0.0001
**Tobacco, alcohol and khat**						
None of them	135 (39.9)	231 (68.3)	Ref.			
One of them	127 (37.6)	82 (24.3)	2.6 (1.87,3.76)	0.0001	2.9 (1.82,4.49)*	0.0001
Two of them	51 (15.1)	19 (5.6)	4.6 (2.6, 8.11)	0.0001	6.3 (3.02,12.98)*	0.0001
All of them	25 (7.4)	6 (1.8)	7.1 (2.85, 17.82)	0.0001	11.6 (3.93,34.53)*	0.0001
**Age categories in years**						
<35	24 (7)	147 (43.4)	Ref.		Ref.	
35-44	46 (14)	62 (18.4)	4.5 (2.56,8.08)	0.0001	3.0 (1.60,5.80)*	0.001
45-54	91 (27)	60 (17.8)	9.3 (5.41,15.95)	0.0001	7.0 (3.97,13.2)*	0.0001
55+	177 (52)	69 (20.4)	15.7 (9.40,26.25)	0.0001	11.0 (6.60,20,91)*	0.0001
**Residency**						
Urban	126 (37.3)	231 (68.3)	Ref.		Ref.	
Rural	212 (62.7)	107 (31.7)	3.6 (2.64, 4.99)	0.001	4.2 (1.04,16.8)*	0.044
**Family monthly income (USD)**						
<35	171 (50.6)	89 (26.3)	Ref.		Ref.	
35-106	130 (38.5)	149 (44.1)	0.45 (0.32,0.64)	0.0001	0.4 (0.27,0.63)*	0.0001
106.6-177	21 (6.2)	71 (21)	0.15 (0.1,0.27)	0.0001	0.2 (0.08,0.30)*	0.0001
>177	16 (4.7)	29 (8.6)	0.30 (0.15,0.56)	0.0001	0.2 (0.09,0.49)*	0.0001
**Tobacco smoking status**						
Never smokers	263 (77.8)	321 (95)	Ref.		Ref.	
Ever smokers	75 (22.2)	17 (5)	5.4 (3.1, 9.34)	0.001	1.3 (1.12,1.60)*	0.047
**Variable**	**Cases (n %)**	**Controls (n %)**	**COR (95%CI)**	**P-value**	**AOR (95% CI)**	**P-value**
**Ever chewed khat**						
No	214 (63.3)	297 (87.9)	Ref.		Ref.	
Yes	124 (36.7)	41 (12.1)	4.2 (2.83, 6.23)	0.0001	4.0 (2.50,6.60)*	0.0001
**Years of chewing khat**	124 (36.7)	41 (12.1)	1.3 (1.08,1.49)	0.003	1.4 (1.21,1.57)*	0.0001
**Spice tea consumption per day**						
≤2 cups	311 (92)	329 (97.3)	Ref.		Ref.	
2+ cups	27 (8.0)	9 (2.7)	3.17 (1.47,6.86)	0.003	4.0 (2.36,16.56)*	0.0001
**Number of coffee consumption per day**						
≤2 cups	169 (50)	197 (58.3)	Ref.		Ref.	
2+ cups	169 (50)	141 (4.7)	1.39 (1.21,1.68)	0.0001	1.2 (1.12,4.64)*	0.007
**Experience of mouth or tongue burn**						
No	224 (66.3)	290 (85.8)	Ref.		Ref.	
Yes	114 (33.7)	48 (14.2)	3.0 (2.1, 4.5)	0.0001	3.1 (2.20,5.83)*	0.004
**Staple diet**						
Teff	201 (60)	230 (68)	Ref.		Ref.	
Sorghum	46 (14)	33 (9.8)	1.6 (1.2, 2.1)	0.001	3.5 (0.74,17.22)	0.11
Maize	32 (8.6)	28 (8.3)	1.3 (1.1, 1.9)	0.04	1.7 (1.69,20.47)*	0.03
Wheat	35 (10.3)	34 (10)	1.2 (0.58, 1.56)	0.053	3.8 (0.91,16.0)	0.07
Barley	10 (3.8)	8 (6.8)	1.4 (1.21, 1.98)	0.001	7.3 (0.13,47.60)	0.07
Others	14 (4.1)	5 (1.5)	3.2 (0.56, 3.9)	0.064	0.8 (0.13,4.39)	0.75
**Ever consumed raw meat**						
No	152 (45)	244 (72.2)	Ref.		Ref.	
Yes	186 (55)	94 (27.8)	3.2 (2.31, 4.38)	0.0001	2.6 (1.75,3.90)*	0.02
**Fruit or vegetable consumption daily**						
No	265 (78.4)	242 (71.6)	Ref.		Ref.	
Yes	73 (21.6)	96 (28.4)	0.39 (0.28,0.55)	0.0001	0.49 (0.32,0.76)*	0.001

*Statistically significant.

## Discussion

Using a case control study design, the researchers investigated the odds/risks factors for oesophageal cancer in Ethiopia. Tobacco users, khat chewers, older people, rural residents, alcohol consumers, red raw meat consumers, and hot beverage users had significantly higher risks/odds of oesophageal cancer, whereas fresh fruit and vegetable consumers and people with better income had significantly lower the risks/odds.

The mean (standard deviation) age of oesophageal cancer patients was 54.3 ± 12.5, whereas the mean (standard deviation) age of controls was 40.9 ± 13.7 years. Our findings suggest that the risk of developing oesophageal cancer increases with age. According to our findings, people over the age of 55 have an 11-fold increased risk of developing oesophageal cancer when compared to people under the age of 35. This corresponds to the followings findings (12, 16–18). Barrett’s esophagus may have become more common in older people, leading to oesophageal adenocarcinoma (19). Consumption of red raw meat has been linked to an increased risk of esophageal cancer (20, 21). These findings were comparable to ours.

Tobacco smoking had increased the odds of oesophageal cancer or smokers were more likely to develop oesophageal cancer than none smokers. This finding is in line with some studies (17, 18, 22–24). The reason could be that the dangerous carcinogens in tobacco cause increased irritation in esophageal cells. It also penetrates the cells of the esophageal epithelium, affecting the cellular part of DNA. As a result, the risk of developing esophageal cancer increases (25).

Ever khat chewing increased the risk of oesophageal cancer development, a finding that is similar to the study reported in reference (17) but unparalleled to that of study (22). A systematic review and meta-analysis supported our finding that there is a link between khat chewing and the development of oesophageal cancer (26). The fact that khat causes oesophageal cancer could be because it causes lesions and inflammations on the buccal mucosa or irritation of the oesophagus, both of which increase the risk of esophageal cancer (27).

Our research found that when these substances are combined, their effects are heightened. Participants who drank alcohol, smoked tobacco, or chewed khat were thus at a higher risk of developing oesophageal cancer. In other words, when people smoke tobacco, drink alcohol, and chew khat together, their risk of developing esophageal cancer rises. Previous research has yielded similar results (28, 29).

In terms of residency, rural residents were found to be at a higher risk of developing oesophageal cancer than urban residents, and similar findings have been reported elsewhere (17, 30). Consumption of hot beverages at high temperatures increased the risk of developing oesophageal cancer (18, 23, 31–35). Our research also discovered that drinking hot spice tea and coffee increased the risk of oesophageal cancer.

Our study found a higher risk of oesophageal cancer among those who consumed red raw meat but a lower risk among those who consumed fresh fruit and vegetables in their dish, similar to other studies (20, 21). Plant-based foods are high in antioxidants, which help to boost our immune system, protect against cancer cells, and reduce our risk of oesophageal cancer (36). This is comparable to our study, which found that the odds of oesophageal cancer decreased by up to 68% and as much as 24%. Consumption of fresh fruits and vegetables on a regular basis reduced the risks of esophageal cancer (24, 29, 37).

The odds/risks of oesophageal cancer development increased among participants who used maize as a staple diet. This is in line with the literature that says maize contains fungi and produces fumonisins that cause cancer-initiating then resulted in oesophageal cancer development (35).

## Strengths and limitations

It is Ethiopia’s first multi-facility study to assess factors associated with the development of oesophageal cancer, and participants were recruited from across the country. Because participants were asked about their previous experiences, our findings could be influenced by recall bias.

## Conclusion

Tobacco smoking, khat chewing, age, residency, hot beverage consumption, and red raw meat consumption were identified as independent positive predictors of oesophageal cancer in this study. Fresh fruit and vegetable consumption, as well as better income, were found to be negative predictors of esophageal cancer. Consumption of fresh fruits and vegetables is encouraged in our diet. Tobacco, khat, hot beverages, red raw meat, and alcohol consumption, on the other hand, were recommended as bad habits to avoid.

## Data availability statement

The raw data supporting the conclusions of this article will be made available by the authors, without undue reservation.

## Ethics statement

The studies involving human participants were reviewed and approved by the Institutional Review Board (IRB) of Addis Ababa University College of Health Sciences with a protocol number of 080/18/SPH. The study followed basic ethical principles of Helsinki declaration for medical research involving human participants (38). Written informed consent for participation was not required for this study in accordance with the national legislation and the institutional requirements.

## Author contributions

All authors contributed from the conception of idea up to data analysis and write up. They also participated in drafting or revising of the article and have agreed on to which journal the article shall be submitted and have given final approval of the version to be published, and agreed to be accountable for all aspects of the work. Specifically, BD was conceptualized the topic of interest, involved in data collection, coding, cleaning, analysis, interpretation of the result unto preparation of the manuscript. FE was involved in proposal development, planning the fieldwork and result section. And MK, MA, and AA were involved in proposal development, data analysis and write up and have participated in critical reviewing of the manuscript.

## Acknowledgments

We would like to express our gratitude to our study participants, data collectors, staff, and administrators at each health facility where participants were recruited for the study.

## Conflict of interest

The authors declare that the research was conducted in the absence of any commercial or financial relationships that could be construed as a potential conflict of interest.

## Publisher’s note

All claims expressed in this article are solely those of the authors and do not necessarily represent those of their affiliated organizations, or those of the publisher, the editors and the reviewers. Any product that may be evaluated in this article, or claim that may be made by its manufacturer, is not guaranteed or endorsed by the publisher.

## Abbreviations

SD, Standard Deviation IQR; Inter Quartile Range USD, United States Dollar DNA; Deoxyribonucleic Acid.
